# Design, Synthesis, and Application of Carbon Dots With Synergistic Antibacterial Activity

**DOI:** 10.3389/fbioe.2022.894100

**Published:** 2022-06-08

**Authors:** Xingwang Qie, Minghui Zan, Ping Gui, Hongyi Chen, Jingkai Wang, Kaicheng Lin, Qian Mei, Mingfeng Ge, Zhiqiang Zhang, Yuguo Tang, Wen-Fei Dong, Yizhi Song

**Affiliations:** ^1^ CAS Key Laboratory of Bio-Medical Diagnostics, Suzhou Institute of Biomedical Engineering and Technology, Chinese Academy of Sciences, Suzhou, China; ^2^ University of Science and Technology of China, Hefei, China

**Keywords:** carbon dots, MurD ligase, photothermal, antibacterial, specificity

## Abstract

The diversity of bacteria and their ability to acquire drug resistance lead to many challenges in traditional antibacterial methods. Photothermal therapies that convert light energy into localized physical heat to kill target microorganisms do not induce resistance and provide an alternative for antibacterial treatment. However, many photothermal materials cannot specifically target bacteria, which can lead to thermal damage to normal tissues, thus seriously affecting their biological applications. Here, we designed and synthesized bacteria-affinitive photothermal carbon dots (BAPTCDs) targeting MurD ligase that catalyzes the synthesis of peptidoglycan (PG) in bacteria. BAPTCDs presented specific recognition ability and excellent photothermal properties. BAPTCDs can bind to bacteria very tightly due to their chiral structure and inhibit enzyme activity by competing with D-glutamic acid to bind to MurD ligases, thus inhibiting the synthesis of bacterial walls. It also improves the accuracy of bacteria treatment by laser irradiation. Through the synergy of biochemical and physical effects, the material offers outstanding antibacterial effects and potentially contributes to tackling the spread of antibiotic resistance and facilitation of antibiotic stewardship.

## Introduction

The variety of pathogenic microorganisms and their rapid mutations can lead to huge challenges in antibacterial treatment (Singer et al., 2016; Zhang et al., 2018a). Multidrug-resistant bacteria are of particular concern (Townsley and Shank, 2017; Maier et al., 2018). Although scientists are still developing new antibiotics, the speed of development lags the speed of mutation in pathogenic microorganisms. Thus, there is an urgent need for new therapies (Yuwen et al., 2018).

Photothermal technology (PTT) has received widespread attention for the treatment of bacterial infections and is a popular nanomedicine. It uses targeted recognition to accumulate photothermal materials near the target tissue and convert light energy into thermal energy *via* an external light source (usually near-infrared light) to kill bacteria (Cheng et al., 2014; Ghosal and Ghosh, 2019). At the heart of PTT is a photothermal material—often nanoparticles such as precious metal materials (Bao et al., 2016), organic dye molecules (Shan et al., 2013), semiconductors (Li et al., 2017), and carbon nanomaterials (Hong et al., 2015). These can convert light energy into heat energy to kill bacteria.

Carbon dots have particularly high application prospects in the field of photothermal materials (Gul et al., 2019; Sun et al., 2019; Tian et al., 2019) because of their excellent physical and chemical properties: good water solubility, excellent biocompatibility, low toxicity, and easy surface functionalization (Sun et al., 2006; Zheng et al., 2015). Carbon dots are used as drugs or drug carriers because of their excellent luminous efficiency that can offer to image while binding to target cells and thus provide real-time monitoring of the treatment effect. However, many photothermal materials cannot specifically target bacteria and can in turn damage normal tissues. In contrast, optimized and functionalized nanomaterials can bind to biological targets.

Peptidoglycan is an important component of bacterial cell walls and offers a rigid structure for growth. It protects the cell from the external environment and high internal osmotic pressure (Lovering et al., 2012). The amide ligases MurC, MurD, MurE, and MurF are involved in its synthesis. These enzymes are excellent antibacterial targets because they are functionally essential for bacterial survival (Miyachiro et al., 2019) and are conserved among all medically relevant bacteria with no counterpart in eukaryotic cells (Kouidmi et al., 2014; Xin et al., 2017). MurD stands out because of its specificity. This specificity is facilitated by a D-type substrate—D-glutamic acid (D-Glu). D-type amino acid molecules can only be metabolized in prokaryotic cells, thus avoiding the effects on eukaryotic cells. Moreover, MurD enzymes have a very strong affinity for D-Glu, and thus, D-Glu derivatives are often used to design inhibitors of the MurD enzyme. To date, however, almost no antibacterial agent can enter bacteria to act on the MurD enzyme because the outer wall of the bacteria is very dense, and inhibitors often fail when coupled with bacterial efflux.

So far, many carbon dots with antibacterial properties have been developed (Das et al., 2020; Raina et al., 2020; Saravanan et al., 2020; Wang et al., 2021; Zhao et al., 2021), but few studies have reported CD-based targeted PTT. Here, novel bacteria-affinitive photothermal carbon dots (BAPTCDs) were prepared for the treatment of bacterial infection via the synergy of their chiral structure and PTT under NIR light irradiation. BAPTCDs were synthesized from D-Glu and o-phenylenediamine via a one-step solvothermal method. The prepared CDs had rapid and effective antibacterial efficacy under NIR light irradiation. The chiral structure gives the CDs a strong affinity to bacteria, thus avoiding damage to host cells. Our findings provide a new strategy for developing antimicrobial materials that can efficiently kill bacteria while avoiding the development of drug resistance, which will greatly relieve the pressure of antibiotic development.

## Experimental Section

### Materials

O-phenylenediamine, D-glutamic acid, and concentrated hydrochloric acid (12 mol L^−1^) were purchased from Energy (Shanghai, China). Dulbecco’s modified Eagle’s medium (DMEM) and trypsin were purchased from Gibco (Shanghai, China). Fetal calf serum (FBS) and 3-(4,5-dimethyl-2-thiazolyl)-2,5-diphenyl-2-H-tetrazolium bromide (MTT) were purchased from Sigma-Aldrich. Luria-Bertani (LB) medium was purchased from MP Biomedical Company (Shanghai, China). HeLa cells were purchased from Beyotime (Shanghai, China). *Escherichia coli* ATCC 700926 and *Staphylococcus aureus* ATCC 29213 were purchased from BioBw (Beijing, China). The microtiter plates were purchased from Corning (Shanghai, China). Confocal Petri dishes with glass bottoms were purchased from MatTek Company (MA, United States). All chemical reagents were used as received without any purification.

### Synthesis of Carbon Dots

O-phenylenediamine (50 mg) and D-Glu (25 mg) were dissolved in 25 ml of HCl aqueous solution (1 mol/L). The solution was transformed into a polytetrafluoroethene-lined autoclave and heated at 200°C in an oven for 3 h. After being cooled to room temperature, the products were centrifuged at 3,000 rpm for 10 min to remove large sediments. To remove the unreacted substrate and muriatic acid, the product was dialyzed against deionized water through a dialysis membrane (500 Da) overnight. Finally, the black powder named BAPTCDs was obtained after freeze-drying for 24 h. Some of the CDs were dissolved in deionized water to 10 mg/ml.

### Characterization

Transmission electron microscopy (TEM) images were obtained by using a JEM-2100 transmission electron microscope at an acceleration voltage of 200 kV. Ultraviolet-visible (UV-Vis) absorption and fluorescence spectra were collected using a UV-Vis V3900H spectrometer and an F4600 spectrometer (HITACHI), respectively. The quantum yield was determined with Rhodamine B as a reference. X-ray photoelectron spectroscopy (XPS) was conducted on a PHI Quantera II electron microscope with a correction voltage of 284.6 eV. Fourier transform infrared (FT-IR) spectra were recorded with a HITACHI Z2012-350 infrared spectrometer. Confocal microscopy images were obtained using an SP5 (Leica) confocal microscope. Scanning electron microscopy images were recorded on an S-4800 (HITACHI) scanning electron microscope. The samples were freeze-dried using an ALPHA1-4/LD plus lyophilizer. The temperature was recorded with a FOTRIC 225s infrared thermal imager.

### Cytotoxicity Testing

We performed MTT experiments with HeLa cells at different concentrations of CDs to investigate the cytotoxicity of carbon dots. The cells were cultured in a 96-well plate at a density of 5,000 cells per well in an incubator (37°C, 5% CO_2_). After being cultured for 24 h, the cell culture medium was replaced with 150 μl of DMEM medium with 10% FBS including carbon dots (0, 50, 100, 300, 500, and 700 μL ml^−1^). Then, 20 μl of 5 mg ml^−1^ MTT reagent was added to each well. After incubation for another 4 h, the medium was removed, and 150 μl of DMSO was added to dissolve the MTT. The resulting mixture was shaken for 10 min at room temperature. The optical density of each well was measured with a microplate reader at 490 nm. Cell viability was evaluated using the following formula:

Cellular activity=ODTreat/ODControl×100%. (1)

Here, OD_Treat_ is the OD value in the presence of carbon dots, and OD_Control_ is the OD value in the absence of carbon dots.

### Test of the Photothermal Capacity

To explore the photothermal properties of BAPTCDs, we prepared BAPTCDs solutions with different concentrations of 100, 200, and 400 μg/ml with PBS and chose PBS only as a control. Here, 1 ml of each solution was placed in a 1.5 ml centrifuge tube, then fixed and continuously irradiated using an 808 nm laser (energy density is 1.5 W/cm^2^) for 10 min. An infrared thermal imager was used to record the temperature change throughout the process (temperature accuracy of 0.1°C).

### Antibacterial Activity Test

Adopted from a previously published protocol (Yu et al., 2021), we tested the antibacterial activity of BAPTCDs via a standard plate counting method. Briefly, *E. coli* ATCC 700926 and *S. aureus* ATCC 29213 were chosen as representative Gram negative and Gram positive bacteria. First, 100 μl of bacterial (10^8^ CFU/ml) suspension and 400 μL of BAPTCD solution (200 μg/ml) were transferred to a centrifuge tube and cultured for 3 h (37°C, 200 rpm) for the full reaction of BAPTCDs and the bacteria. Then, the mixture was immediately either treated with NIR (808 nm, 1.5 W/cm^2^) for 10 min or with no irradiation. The BAPTCD solution was replaced with PBS in the control groups. The number of bacteria was counted by plating 100 μl of 10-fold serial dilutions onto the LB agar plates. All plates were cultured at 37°C for 16 h, and the bacterial viability was calculated according to the equation:

$$\mathrm{Bacterial}\;\mathrm{viability}=N_{e}/N_{c}\times 100\%,$$ (2)

where N_c_ is colonies of bacteria treated by PBS, and N_e_ is colonies of bacteria treated by different methods.

To examine the binding ability of BAPTCDs and the morphology change of bacteria after the treatment, confocal imaging and SEM were conducted. The bacterial solution in the centrifuge tube was centrifuged and washed three times with sterilized PBS, and then, the bacteria were transferred to confocal Petri dishes to observe their fluorescence with a confocal microscope. Subsequently, 2.5% glutaraldehyde solution was added to the bacterial solution for 2 h to fix the bacteria. After being washed for three times, the bacteria were dehydrated by adding 200 μl of different concentrations of ethanol in a gradient (30%, 50%, 70%, 90%, and 100%) for 15 min each time. After the samples were dried, the bacterial morphology was observed by SEM.

## Results and Discussion

### Synthesis and Characterization of Bacteria-Affinitive Photothermal Carbon Dots

**FIGURE 1:**
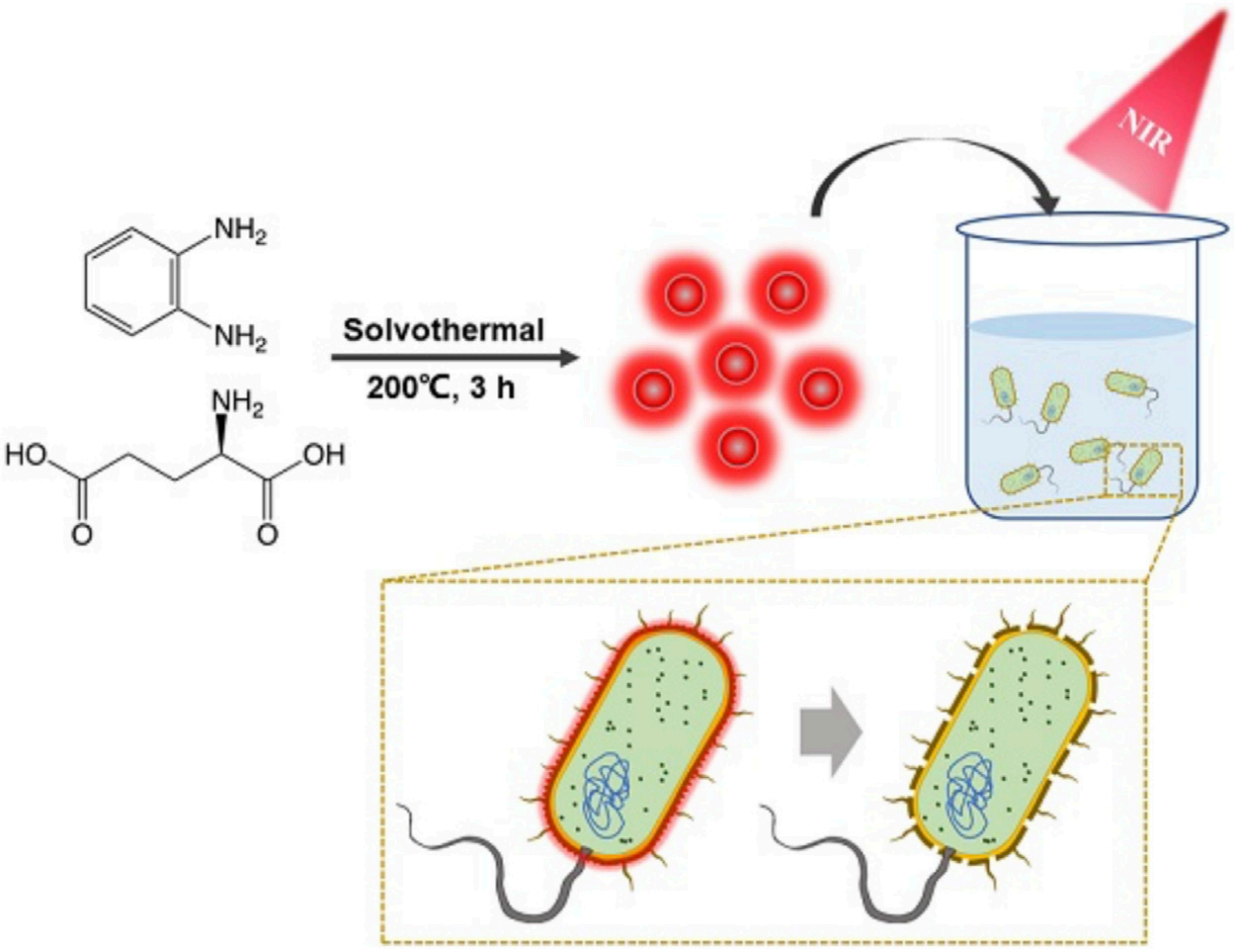
Schematic illustration of synthesis of the BAPTCD mechanism of bacteria targeting and photothermal ablation of BAPTCDs upon laser irradiation.

We investigated the recent research on antibacterial carbon dots and found that few photothermal carbon dots can be targeted to bacteria, which will pose great challenges to their application in humans (Table 1). Therefore, we chose D-Glu and o-phenylenediamine as precursors and synthesize a new type of nitrogen-doped carbon dots (BAPTCDs) *via* a one-step solvothermal method. The manufacturing process is shown in Figure 1. We optimized the experimental conditions to improve the quantum yield to 38% (reactant ratio of 1:2, 200°C, and reaction time of 3 h; Supplementary Tables S1, S2).

**TABLE 1:** Antibacterial carbon dots with the chiral structure or PTT in recent years.

Number	Synthesis method	Size	PL color	Applications	PTT/chiral	Reference
1	Pyrolysis	3 nm	Blue	Antimicrobial agent antibiotic carrier fluorescent probe	Chiral	Xin et al. (2017)
2	Microwave	∼10 nm	Blue	Antimicrobial agent	Chiral	Victoria et al. (2020)
3	Microwave	5 nm	—	Antibacterial sensing drug delivery	PTT	Lu et al. (2018)
4	Hydrothermal	3 nm	Blue	Antibacterial therapy	PTT	Yan et al. (2021)
5	Hydrothermal	∼1.3 nm	Green	Antibacterial therapy	PTT	Behzadpour et al. (2019)
6	Hydrothermal	190 ± 20 nm	—	Antibacterial therapy	PTT	Liu et al. (2021)
7	Solvothermal	4.2 nm	Blue, green, and red	Antimicrobial agent bioimaging	PTT	Chu et al. (2021)
8	Solvothermal	170 nm	—	Antibacterial agent	PTT	Wu et al. (2018)
9	Solvothermal	3–5 nm	Red	Antimicrobial agent	PTT and chiral	This study

**FIGURE 2:**
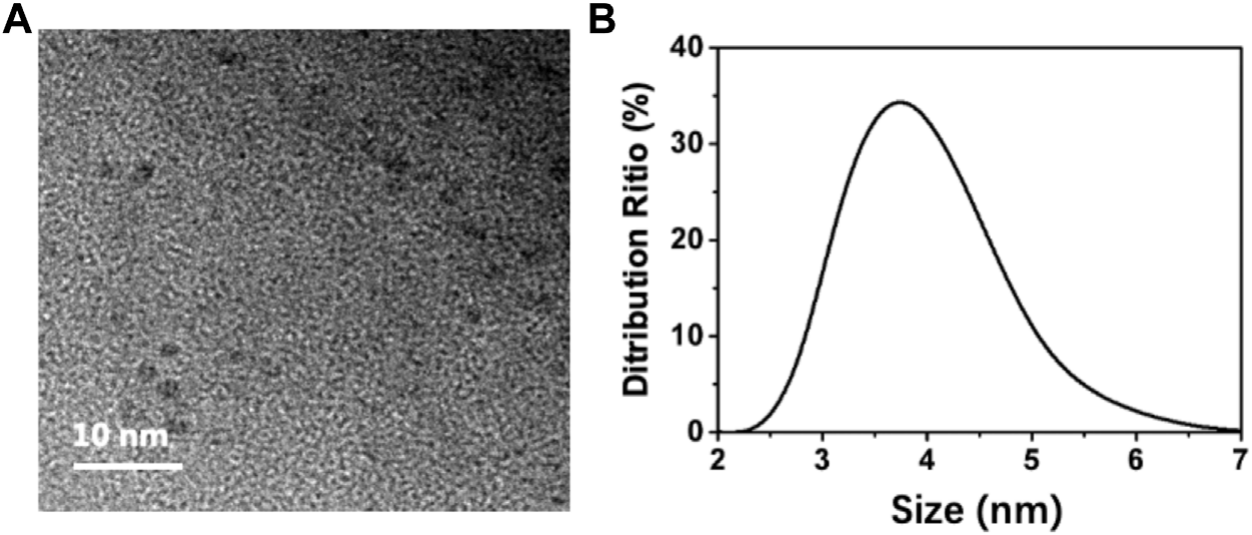
Morphology of BAPTCDs. **(A)** TEM image of BAPTCDs (scale bar: 10 nm). **(B)** Dynamic light-scattering detection of BAPTCDs.

To analyze the morphology of the BAPTCDs we performed TEM and DLS. Figure 2A shows the TEM image: the carbon dots were spherical and uniformly distributed with a size of 3 nm. DLS showed that the BAPTCD size distribution was normal with the peak between 3 and 5 nm (Figure 2B). DLS was performed in the solution, and the particles will stretch and deform after absorbing water; thus, the DLS data are consistent with TEM.

**FIGURE 3:**
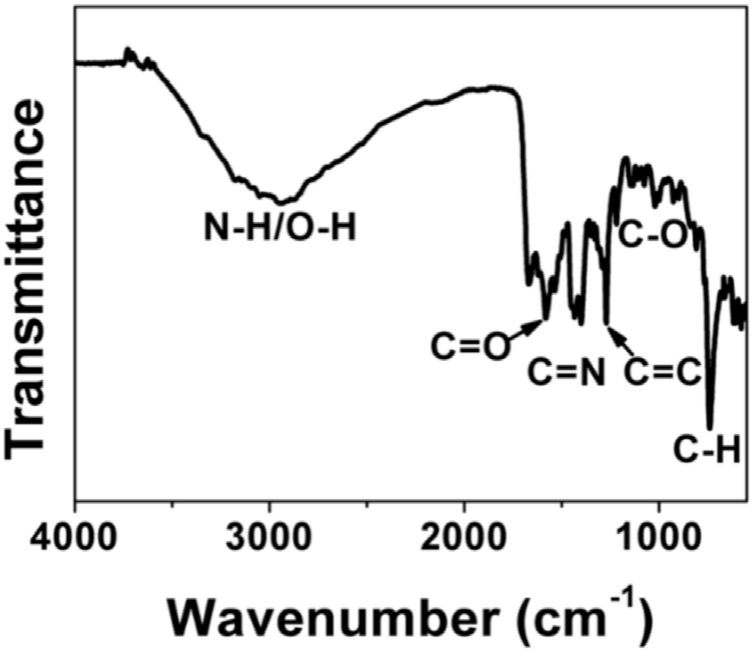
FT-IR spectrum of BAPTCDs.

**FIGURE 4:**
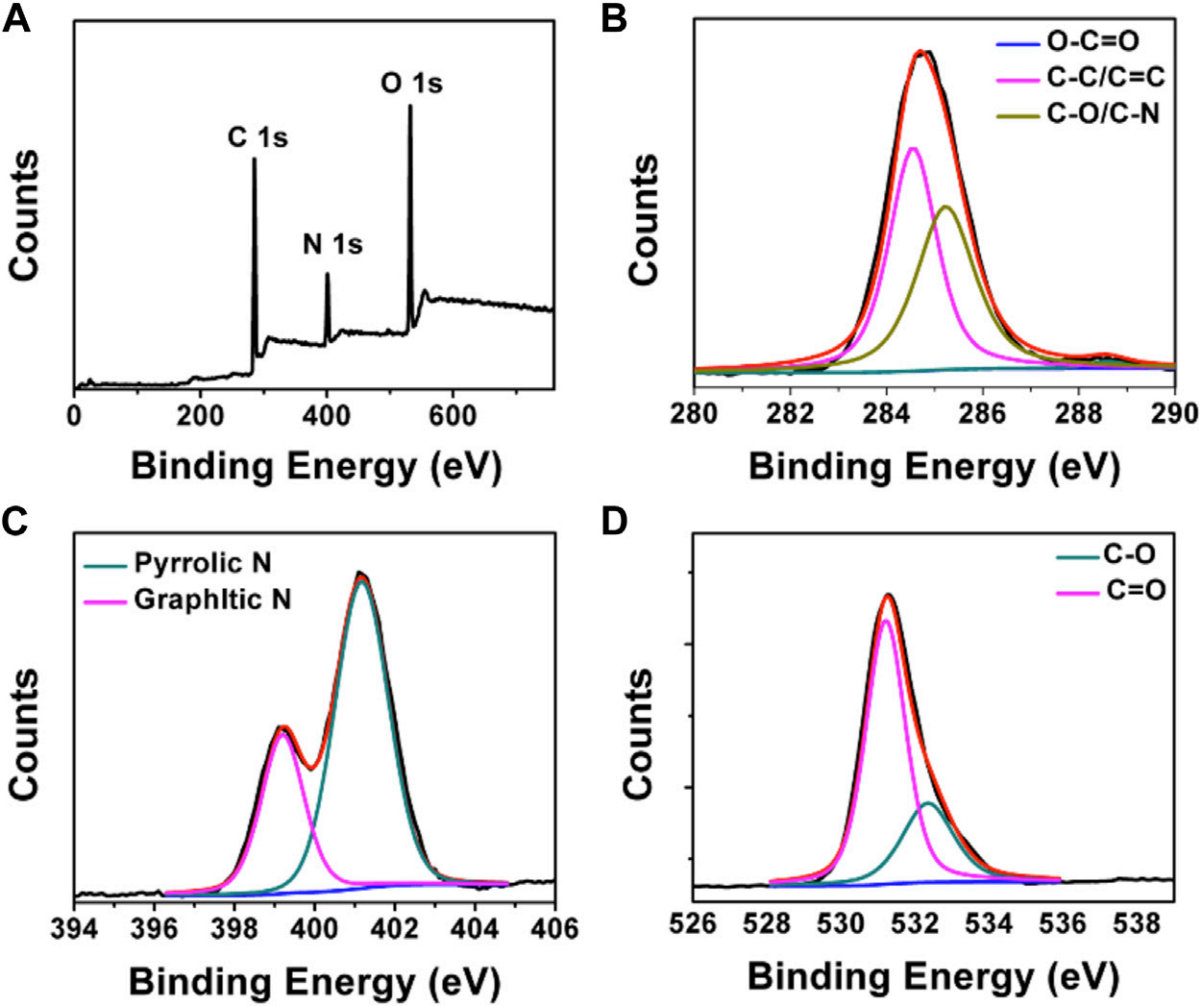
**(A)** XPS spectrum of BAPTCDs and **(B–D)** the high-resolution XPS spectra of C 1s, N 1s, and O 1s, respectively.

FT-IR and XPS measurements were performed to investigate the BAPTCD composition. Figure 3 shows the FT-IR spectrum of the prepared CDs. The broad absorption peak at 3,444 cm^−1^ is attributed to N-H and O-H, indicating its excellent solution properties. The small peaks at 1,677 cm^−1^, 1,574 cm^−1^, 1,503 cm^−1^, and 1,131 cm^−1^ were assigned to C=O, C=O, C=C, and C-O stretching vibrations. The absorption peak at 675 cm^−1^ corresponds to the stretching vibration of the functional group C-H. These functional groups indicated strong nitrogen doping. The XPS survey spectra of BAPTCDs (Figure 4A) showed the presence of C, N, and O with percentages of 57.2%, 18.4%, and 24.4%, respectively; the corresponding C 1s, N 1s, and O 1s peaks were located at 284.7, 401.1, and 531.3 eV, respectively. The C 1s spectrum of BAPTCDs (Figure 4B) is composed of three peaks at 284.7, 401.1, and 531.3 eV, which suggests the presence of C-C/C=C, C-O/C-N, and O-C=O, respectively. The N 1s spectrum of BAPTCDs (Figure 4C) can be divided into two obvious peaks at 399.2 and 401.2 eV, which is consistent with the chemical bonds of graphitic N and pyrrolic N. The O 1s spectrum of BAPTCDs (Figure 4D) can also be deconvoluted into two distinct peaks at 531.2 and 532.4 eV, consistent with C=O and C-O, respectively. These structural features suggest the successful doping of N; the functional groups were remarkably consistent with the FT-IR spectrum.

### Optical Property of Bacteria-Affinitive Photothermal Carbon Dots

**FIGURE 5:**
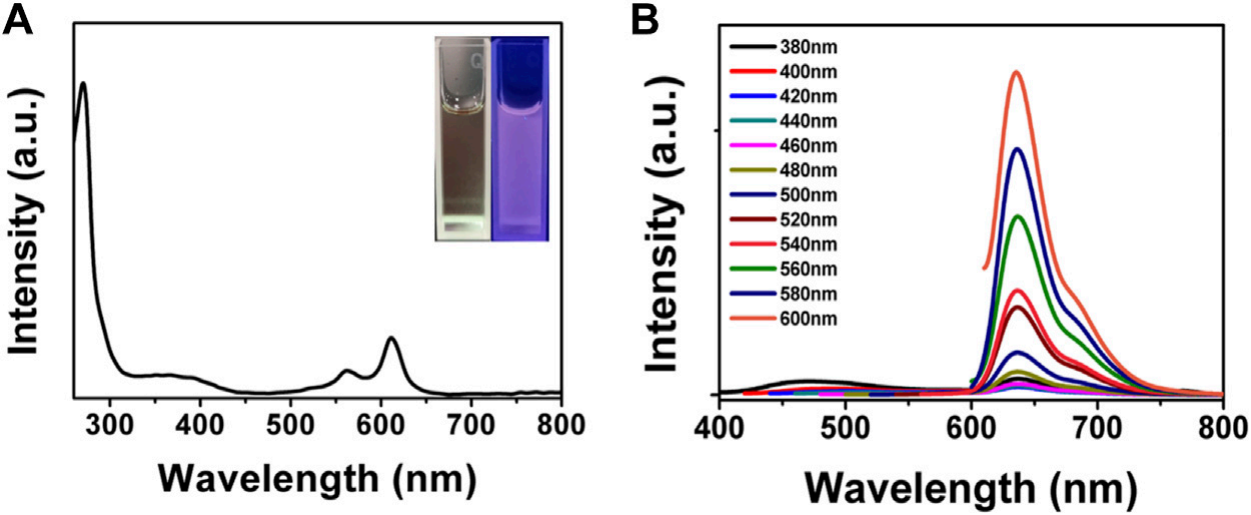
Optical property of BAPTCDs. **(A)** UV-vis spectrum of BAPTCDs. The insets are the photograph of BAPTCD aqueous solution under visible light (left) and UV light with the wavelength of 365 nm (right). **(B)** Fluorescence emission spectra of BAPTCDs.

We next investigated the optical properties of BAPTCDs. The UV-vis spectrum of BAPTCDs showed two absorption peaks centered at 560 and 612 nm (Figure 5A). This might be because the high temperature increases oxidation on the surface of the carbon dots and decreases the band gap between the lowest unoccupied molecular orbital (LUMO) and the highest occupied molecular orbital (HOMO), thus causing a red-shift in the carbon dots (Song et al., 2015). The light blue BAPTCD aqueous solution emitted light red fluorescence under visible light and emitted bright red fluorescence when irradiated with a 365 nm lamp (Figure 5 inset). Figure 5B shows the fluorescence emission spectra of BAPTCDs. Although the excitation wavelength increased from 380 to 600 nm, and the optimal emission wavelength remained at 639 nm. Excitation-development photoluminescence behaviors common in carbon dot materials were not observed, which may be due to the relatively uniform particle size and special surface functional groups of BAPTCDs (Li et al., 2010; Zhang et al., 2016).

### Photothermal Properties of Bacteria-Affinitive Photothermal Carbon Dots

**FIGURE 6:**
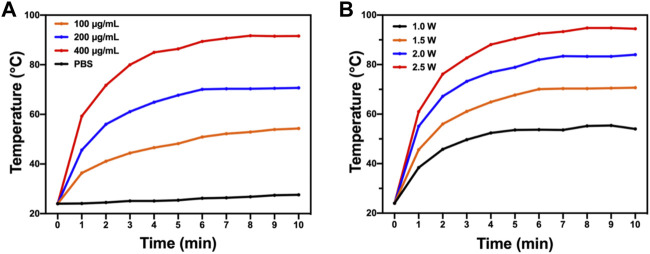
Photothermal property of BAPTCDs. **(A)** Temperature change of BAPTCDs aqueous with different concentrations upon 10 min of 808 nm laser irradiation (power density = 1.5 W/cm^2^). **(B)** BAPTCDs at the concentration of 200 μg/ml with a series of power densities of 808 nm laser irradiation.

PTT is known for its deep penetration and effective photothermal conversion ability which can cause irreversible damage to biological tissues and cells and quickly kill bacteria. An 808-nm near-infrared laser was used to test the photothermal performance of BAPTCDs. Figure 6A shows that the temperature of the BAPTCD solution with different concentrations rapidly increased and stabilized within 10 min. The maximum temperature of BAPTCD solutions with concentrations of 100, 200, and 400 μg/ml under laser irradiation increased to 52.2°C, 70.3°C, and 90.7°C, respectively. However, there was no obvious temperature change in the PBS solution, which indicates that BAPTCDs have a fast and efficient ability to convert near-infrared light energy into heat. Figure 6B shows the temperature change of the BAPTCD solution (200 μg/ml) exposed to an 808-nm laser at various laser power densities (1.0, 1.5, 2.0, and 2.5 W/cm^2^). The results showed that a higher power density can heat the BAPTCD solution to a higher temperature. Considering that high temperatures can damage the host cells, we used a concentration of 200 μg/ml and the power density of 1.5 W/cm^2^ to test the CDs’ antibacterial activity.

### Cytotoxicity test of Bacteria-Affinitive Photothermal Carbon Dots

We chose HeLa cells to test the toxicity of BAPTCDs to host cells with an MTT assay. As shown in Supplementary Figure S1, the MTT assay revealed that the average cell viability is higher than 85% even with BAPTCD concentration reaching 400 μg/ml. These results suggested that BAPTCDs have low toxicity to HeLa cells at a relatively high concentration. Supplementary Figure S2 showed that the BAPTCDs cannot bind to HeLa cells. This may be due to their chiral structure (Kouidmi et al., 2014; Xin et al., 2017; Zhang et al., 2018b; Zhang et al., 2020), which contributes to the low toxicity of BAPTCDs to cells. Thus, BAPTCDs will be a very safe material for human use.

### Antibacterial Activity of Bacteria-Affinitive Photothermal Carbon Dots

**FIGURE 7:**
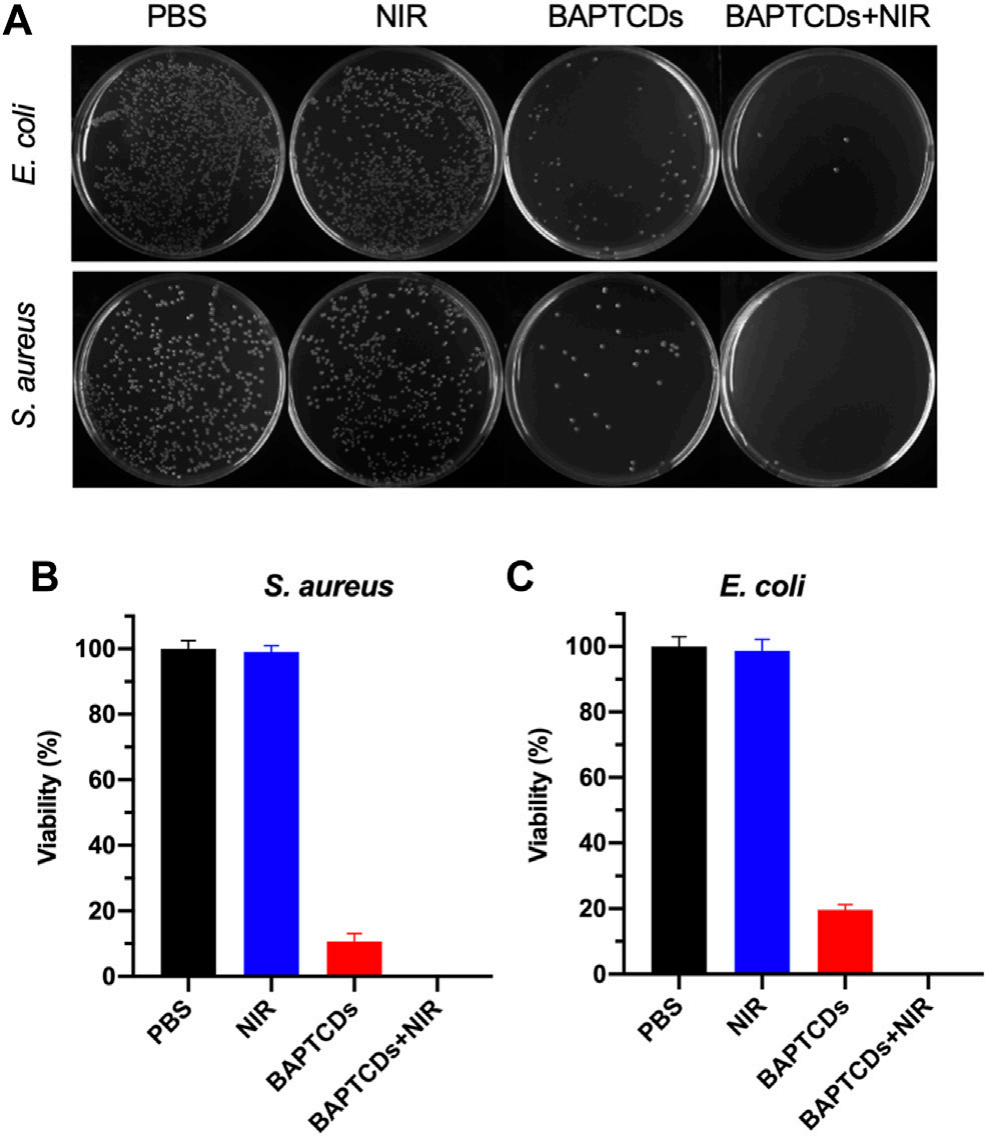
**(A)** Photographic images of the colonies of *E. coli* ATCC 700926 and *S. aureus* ATCC 29213 treated by PBS, BAPTCDs, BAPTCDs with NIR (power density = 1.5 W/cm^2^), and NIR only by a standard plate count method. The concentration of BAPTCDs was 200 μg/ml. Bacterial viability of *E. coli* ATCC 700926 **(B)** and *S. aureus* ATCC 29213 **(C)** were obtained by the colony-forming count method. (Error bars represent the standard deviation of at least three independent experiments.).

To test the antibacterial activity of BAPTCDs, we treated the bacteria with carbon dots and lasers and evaluated the antibacterial efficiency via a standard plate counting method. Figure 7 shows that 80.33% of *E*. *coli* and 89.27% of *S*. *aureus* were killed by BAPTCDs without NIR, which may contribute to its inhibition of the MurD protein. When irradiated with NIR (808 nm, 1.5 W/cm^2^), only 3.67% of *E. coli* survived and all of the *S. aureus* were killed due to the rapidly rising temperature.

**FIGURE 8:**
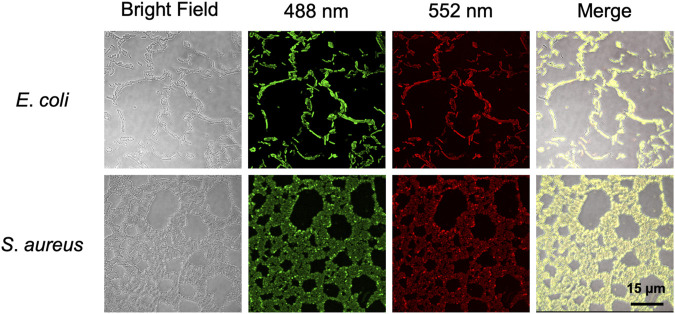
Confocal microscopy images of *E. coli* ATCC 700926 and *S. aureus* ATCC 29213 treated with BAPTCDs. From left to right: bright field, fluorescent image excited with a 488 and 552 nm laser, and overlap, respectively.

To further explore whether BABTCDs bind to bacteria as we designed, we performed fluorescence imaging to observe the position of BAPTCDs. BAPTCDs showed a strong affinity for bacteria (Figure 8) as all bacteria were wrapped by carbon dots and emitted bright fluorescence under laser irradiation. The affinity increases the spatial accuracy of the antibacterial material and reduces the damage to host cells.

**FIGURE 9:**
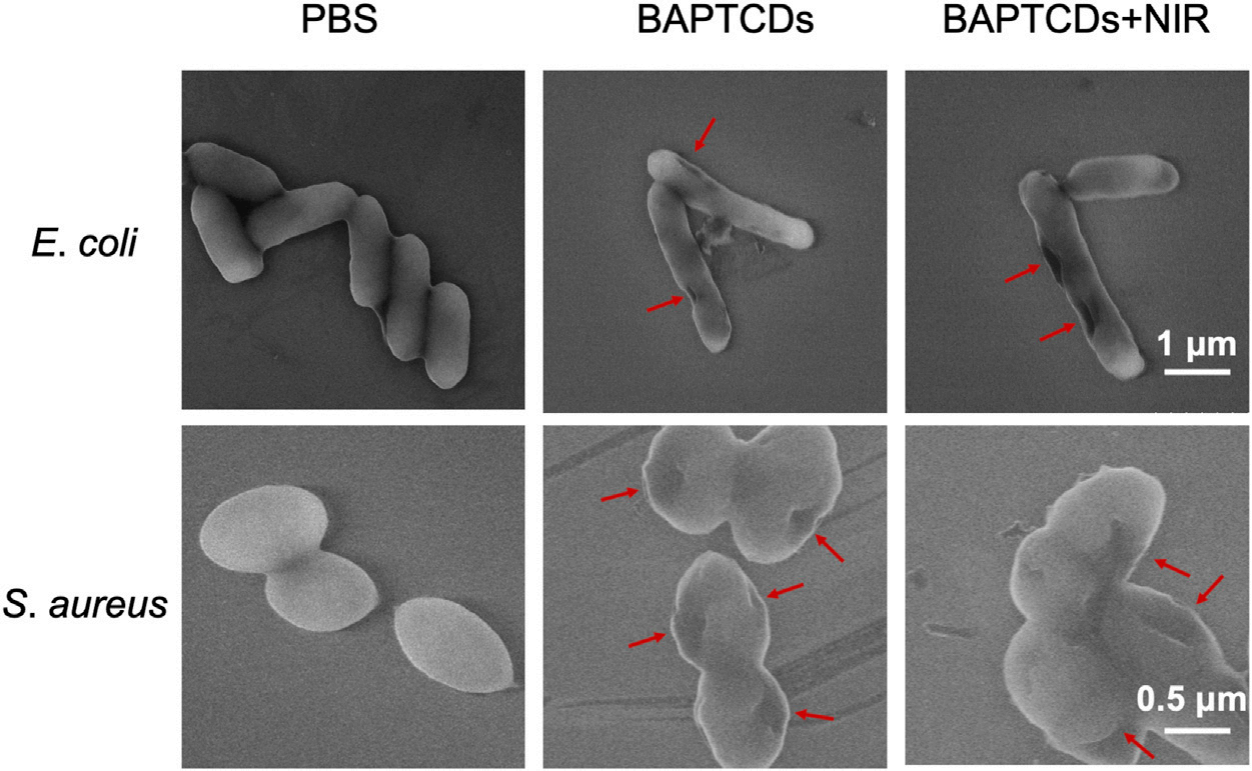
SEM images of *E. coli* ATCC 700926 and *S. aureus* ATCC 29213 were treated by using BAPTCDs at 200 μg/ml with or without NIR. (808 nm with the power density of 1.5 W/cm^2^). The red arrow shows where the bacterial cell wall has broken.

To assess the effect of cell wall damage by BAPTCDs, the morphological changes of bacteria after the treatment were observed under SEM. The SEM images (Figure 9) showed that bacteria without BAPTCD treatment were in good condition with a complete cell wall while the cavities occurred on the cell surface when treated with BAPTCDs even without NIR irradiation. This was consistent with a previous study in which the researchers proposed that carbon dots with a chiral structure may penetrate the bacterial cell wall and specifically bind to cytoplasmic proteins, resulting in damage to the cell wall (Xin et al., 2017). When exposed to NIR, obvious lethal effects occurred. The cell walls were badly damaged, leading to bacterial death. This approach mainly uses physical action to sterilize to avoid the generation of drug resistance. Thus, it may be an effective substitute for antibiotics.

## Conclusion

In summary, we synthesized novel chiral biomolecule functionalized CDs (BAPTCDs) *via* a one-step solvothermal method. BAPTCDs were designed to specifically bind to bacteria and achieve synergistic antibacterial effects. BAPTCDs have extraordinary photothermal properties and can damage the cell wall of bacteria *via* a rapid temperature rise. With the ability of targeted binding to bacteria, BAPTCDs showed little cytotoxicity to human cell lines but an antibacterial efficiency of over 99%. Therefore, the surface chiral design of nanomaterials offers us a promising approach to improve the bactericidal efficacy of materials with minimal cytotoxicity to normal tissues.

## Data Availability

The original contributions presented in the study are included in the article/Supplementary Material; further inquiries can be directed to the corresponding authors.

## References

[B1] Bao Z. Liu X. Liu Y. Liu H. Zhao K. (2016). Near-infrared Light-Responsive Inorganic Nanomaterials for Photothermal Therapy. Asian J. Pharm. Sci. 11 (3), 349–364. 10.1016/j.ajps.2015.11.123

[B2] Behzadpour N. Sattarahmady N. Akbari N. (2019). Antimicrobial Photothermal Treatment of pseudomonas Aeruginosa by a Carbon Nanoparticles-Polypyrrole Nanocomposite. J. Biomed. Phys. Eng. 9 (6), 661. 10.31661/jbpe.v0i0.1024 32039097 PMC6943850

[B3] Cheng L. Wang C. Feng L. Yang K. Liu Z. (2014). Functional Nanomaterials for Phototherapies of Cancer. Chem. Rev. 114 (21), 10869–10939. 10.1021/cr400532z 25260098

[B4] Chu X. Zhang P. Wang Y. Sun B. Liu Y. Zhang Q. (2021). Near-infrared Carbon Dot-Based Platform for Bioimaging and Photothermal/photodynamic/quaternary Ammonium Triple Synergistic Sterilization Triggered by Single NIR Light Source. Carbon 176, 126–138. 10.1016/j.carbon.2021.01.119

[B5] Das P. Maruthapandi M. Saravanan A. Natan M. Jacobi G. Banin E. (2020). Carbon Dots for Heavy-Metal Sensing, pH-Sensitive Cargo Delivery, and Antibacterial Applications. ACS Appl. Nano Mater. 3 (12), 11777–11790. 10.1021/acsanm.0c02305

[B6] Ghosal K. Ghosh A. (2019). Carbon Dots: The Next Generation Platform for Biomedical Applications. Mater. Sci. Eng. C 96, 887–903. 10.1016/j.msec.2018.11.060 30606603

[B7] Gul A. R. Le T. N. Kim M. W. Kailasa S. K. Oh K. T. Park T. J. (2019). One-pot Synthesis of Carbon Dots with Intrinsic Folic Acid for Synergistic Imaging-Guided Photothermal Therapy of Prostate Cancer Cells. Biomaterials Sci. 7 (12), 5187–5196. 10.1039/c9bm01228a 31588457

[B8] Hong G. Diao S. Antaris A. L. Dai H. (2015). Carbon Nanomaterials for Biological Imaging and Nanomedicinal Therapy. Chem. Rev. 115 (19), 10816–10906. 10.1021/acs.chemrev.5b00008 25997028

[B9] Kouidmi I. Levesque R. C. Paradis-Bleau C. (2014). The Biology of Mur Ligases as an Antibacterial Target. Mol. Microbiol. 94 (2), 242–253. 10.1111/mmi.12758 25130693

[B10] Li A. Li X. Yu X. Li W. Zhao R. An X. (2017). Synergistic Thermoradiotherapy Based on PEGylated Cu 3 BiS 3 Ternary Semiconductor Nanorods with Strong Absorption in the Second Near-Infrared Window. Biomaterials 112, 164–175. 10.1016/j.biomaterials.2016.10.024 27768971

[B11] Li H. He X. Kang Z. Huang H. Liu Y. Liu J. (2010). Water-Soluble Fluorescent Carbon Quantum Dots and Photocatalyst Design. Angew. Chem. Int. Ed. 49 (26), 4430–4434. 10.1002/anie.200906154 20461744

[B12] Liu Z. Zhao X. Yu B. Zhao N. Zhang C. Xu F.-J. (2021). Rough Carbon-Iron Oxide Nanohybrids for Near-Infrared-II Light-Responsive Synergistic Antibacterial Therapy. ACS Nano 15 (4), 7482–7490. 10.1021/acsnano.1c00894 33856198

[B13] Lovering A. L. Safadi S. S. Strynadka N. C. J. (2012). Structural Perspective of Peptidoglycan Biosynthesis and Assembly. Annu. Rev. Biochem. 81, 451–478. 10.1146/annurev-biochem-061809-112742 22663080

[B14] Lu Y. Li L. Li M. Lin Z. Wang L. Zhang Y. (2018). Zero-dimensional Carbon Dots Enhance Bone Regeneration, Osteosarcoma Ablation, and Clinical Bacterial Eradication. Bioconjugate Chem. 29 (9), 2982–2993. 10.1021/acs.bioconjchem.8b00400 PMC6380686 29986578

[B15] Maier L. Pruteanu M. Kuhn M. Zeller G. Telzerow A. Anderson E. E. (2018). Extensive Impact of Non-antibiotic Drugs on Human Gut Bacteria. Nature 555 (7698), 623–628. 10.1038/nature25979 29555994 PMC6108420

[B16] Miyachiro M. M. Granato D. Trindade D. M. Ebel C. Paes Leme A. F. Dessen A. (2019). Complex Formation between Mur Enzymes from Streptococcus Pneumoniae. Biochemistry 58 (30), 3314–3324. 10.1021/acs.biochem.9b00277 31264408

[B17] Raina S. Thakur A. Sharma A. Pooja D. Minhas A. P. (2020). Bactericidal Activity of Cannabis Sativa Phytochemicals from Leaf Extract and Their Derived Carbon Dots and Ag@Carbon Dots. Mater. Lett. 262, 127122. 10.1016/j.matlet.2019.127122

[B18] Saravanan A. Maruthapandi M. Das P. Ganguly S. Margel S. Luong J. H. T. (2020). Applications of N-Doped Carbon Dots as Antimicrobial Agents, Antibiotic Carriers, and Selective Fluorescent Probes for Nitro Explosives. ACS Appl. Bio Mater. 3 (11), 8023–8031. 10.1021/acsabm.0c01104 35019541

[B19] Shan G. Weissleder R. Hilderbrand S. A. (2013). Upconverting Organic Dye Doped Core-Shell Nano-Composites for Dual-Modality NIR Imaging and Photo-Thermal Therapy. Theranostics 3 (4), 267–274. 10.7150/thno.5226 23606913 PMC3630527

[B20] Singer M. Deutschman C. S. Seymour C. W. Shankar-Hari M. Annane D. Bauer M. (2016). The Third International Consensus Definitions for Sepsis and Septic Shock (Sepsis-3). Jama 315 (8), 801–810. 10.1001/jama.2016.0287 26903338 PMC4968574

[B21] Song Y. Zhu S. Zhang S. Fu Y. Wang L. Zhao X. (2015). Investigation from Chemical Structure to Photoluminescent Mechanism: a Type of Carbon Dots from the Pyrolysis of Citric Acid and an Amine. J. Mater. Chem. C 3 (23), 5976–5984. 10.1039/c5tc00813a

[B22] Sun S. Chen J. Jiang K. Tang Z. Wang Y. Li Z. (2019). Ce6-Modified Carbon Dots for Multimodal-Imaging-Guided and Single-NIR-Laser-Triggered Photothermal/Photodynamic Synergistic Cancer Therapy by Reduced Irradiation Power. ACS Appl. Mater. Interfaces 11 (6), 5791–5803. 10.1021/acsami.8b19042 30648846

[B23] Sun Y.-P. Zhou B. Lin Y. Wang W. Fernando K. A. S. Pathak P. (2006). Quantum-sized Carbon Dots for Bright and Colorful Photoluminescence. J. Am. Chem. Soc. 128 (24), 7756–7757. 10.1021/ja062677d 16771487

[B24] Tian L. Tao L. Li H. Zhao S. Zhang Y. Yang S. (2019). Hollow Mesoporous Carbon Modified with cRGD Peptide Nanoplatform for Targeted Drug Delivery and Chemo-Photothermal Therapy of Prostatic Carcinoma. Colloids Surfaces A Physicochem. Eng. Aspects 570, 386–395. 10.1016/j.colsurfa.2019.03.030

[B25] Townsley L. Shank E. A. (2017). Natural-product Antibiotics: Cues for Modulating Bacterial Biofilm Formation. Trends Microbiol. 25 (12), 1016–1026. 10.1016/j.tim.2017.06.003 28688575 PMC5701842

[B26] Victoria F. Manioudakis J. Zaroubi L. Findlay B. Naccache R. (2020). Tuning Residual Chirality in Carbon Dots with Anti-microbial Properties. RSC Adv. 10 (53), 32202–32210. 10.1039/d0ra05208f 35518167 PMC9056545

[B27] Wang B. Song H. Qu X. Chang J. Yang B. Lu S. (2021). Carbon Dots as a New Class of Nanomedicines: Opportunities and Challenges. Coord. Chem. Rev. 442, 214010. 10.1016/j.ccr.2021.214010

[B28] Wu Q. Wei G. Xu Z. Han J. Xi J. Fan L. (2018). Mechanistic Insight into the Light-Irradiated Carbon Capsules as an Antibacterial Agent. ACS Appl. Mater. Interfaces 10 (30), 25026–25036. 10.1021/acsami.8b04932 29989399

[B29] Xin Q. Liu Q. Geng L. Fang Q. Gong J. R. (2017). Chiral Nanoparticle as a New Efficient Antimicrobial Nanoagent. Adv. Healthc. Mater 6 (4). 10.1002/adhm.201601011 28026134

[B30] Yan H. Zhang B. Zhang Y. Su R. Li P. Su W. (2021). Fluorescent Carbon Dot-Curcumin Nanocomposites for Remarkable Antibacterial Activity with Synergistic Photodynamic and Photothermal Abilities. ACS Appl. Bio Mater. 4 (9), 6703–6718. 10.1021/acsabm.1c00377 35006973

[B31] Yu Z. Jiang L. Liu R. Zhao W. Yang Z. Zhang J. (2021). Versatile Self-Assembled MXene-Au Nanocomposites for SERS Detection of Bacteria, Antibacterial and Photothermal Sterilization. Chem. Eng. J. 426. 10.1016/j.cej.2021.131914

[B32] Yuwen L. Sun Y. Tan G. Xiu W. Zhang Y. Weng L. (2018). MoS2@polydopamine-Ag Nanosheets with Enhanced Antibacterial Activity for Effective Treatment of Staphylococcus aureus Biofilms and Wound Infection. Nanoscale 10 (35), 16711–16720. 10.1039/c8nr04111c 30156245

[B33] Zhang L. Wang Y. Wang J. Wang Y. Chen A. Wang C. (2018). Photon-responsive Antibacterial Nanoplatform for Synergistic Photothermal-/pharmaco-Therapy of Skin Infection. ACS Appl. Mater. Interfaces 11 (1), 300–310. 10.1021/acsami.8b18146 30520301

[B34] Zhang M. Hu L. Wang H. Song Y. Liu Y. Li H. (2018). One-step Hydrothermal Synthesis of Chiral Carbon Dots and Their Effects on Mung Bean Plant Growth. Nanoscale 10 (26), 12734–12742. 10.1039/c8nr01644e 29946587

[B35] Zhang M. Zhang H. Feng J. Zhou Y. Wang B. (2020). Synergistic Chemotherapy, Physiotherapy and Photothermal Therapy against Bacterial and Biofilms Infections through Construction of Chiral Glutamic Acid Functionalized Gold Nanobipyramids. Chem. Eng. J. 393. 10.1016/j.cej.2020.124778

[B36] Zhang Z. Pan Y. Fang Y. Zhang L. Chen J. Yi C. (2016). Tuning Photoluminescence and Surface Properties of Carbon Nanodots for Chemical Sensing. Nanoscale 8 (1), 500–507. 10.1039/c5nr06534h 26676688

[B37] Zhao D. Liu X. Zhang R. Huang X. Xiao X. (2021). Facile One-Pot Synthesis of Multifunctional Protamine Sulfate-Derived Carbon Dots for Antibacterial Applications and Fluorescence Imaging of Bacteria. New J. Chem. 45 (2), 1010–1019. 10.1039/d0nj04458j

[B38] Zheng X. T. Ananthanarayanan A. Luo K. Q. Chen P. (2015). Glowing Graphene Quantum Dots and Carbon Dots: Properties, Syntheses, and Biological Applications. Small 11 (14), 1620–1636. 10.1002/smll.201402648 25521301

