# Supplementary material for: Design, Synthesis, and Application of Carbon Dots With Synergistic Antibacterial Activity
Source: Front Bioeng Biotechnol. 2022 Jun 8;10:894100. doi: 10.3389/fbioe.2022.894100 (PMC9213729; doi:10.3389/fbioe.2022.894100)
Supplement: Supplementary file 1 [file DataSheet1.docx]

Supplementary Materials

**Design, synthesis, and application of carbon dots with synergistic antibacterial** **activity**

Xingwang Qie ^a,1^, Minghui Zan^a^, Ping Gui ^a^, Hongyi Chen^a,b^, Jingkai Wang^a,b^, Kaicheng Lin^a^, Qian Mei^a^, Mingfeng Ge^a^, Zhiqiang Zhang^a^, Yuguo Tang^a^, Wen-Fei Dong ^[[1]](#footnote-1)^*^a,b^, Yizhi Song ^[[2]](#footnote-2)^*^a,b^

^a^ CAS Key Laboratory of Bio-Medical Diagnostics, Suzhou Institute of Biomedical Engineering and Technology, Chinese Academy of Sciences, Suzhou 215163, PR China

^b^ University of Science and Technology of China, Hefei 230026, PR China

**Table S1**. Fluorescence quantum yield of BAPTCDs prepared from different reaction ratios for 3h.

| Sample | m _D-Glu_:m _o-phenylenediamine_ | QY(%) |
| --- | --- | --- |
| 1 | 2:1 | 26 |
| 2 | 1:1 | 30 |
| 3 | 1:2 | 38 |

**Table S2.** Fluorescence quantum yield of BAPTCDs prepared from different reaction time with the reaction ratio of 1:2.

| Sample | Reaction time (h) | QY(%) |
| --- | --- | --- |
| 1 | 3 | 38 |
| 2 | 6 | 22 |
| 3 | 9 | 15 |


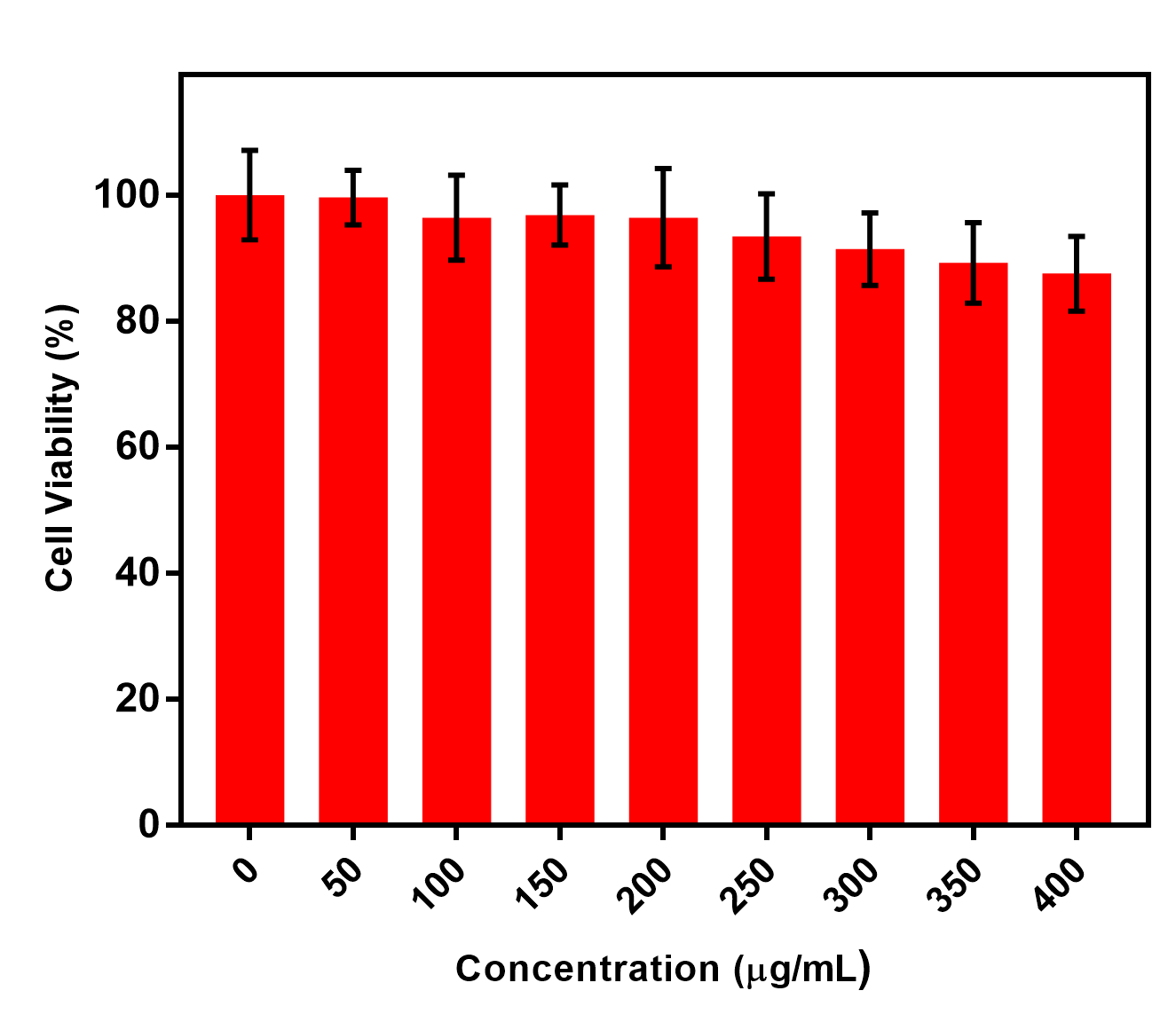


**Figure S1**. Cell viability assay of HeLa cells treated with different concentrations of BAPTCDs. ( Error bars represent the standard deviation of at least three independent experiments.)


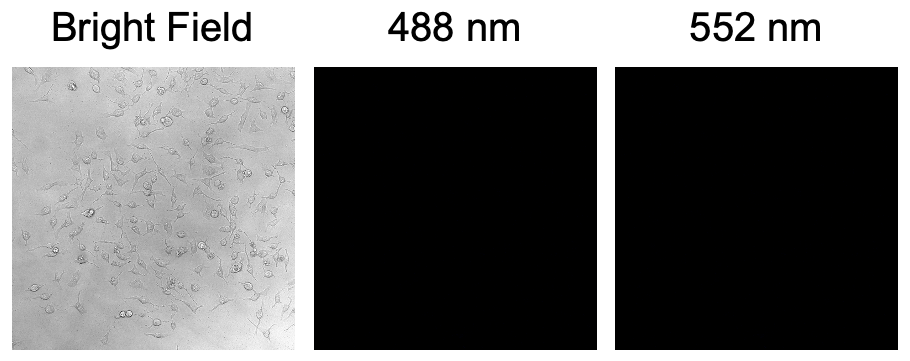


**Figure S2**. Confocal microscopy images of Hela cells treated with BAPTCDs. From left to right: bright field, fluorescent image excited with a 488 nm and 552 nm laser and overlap, respectively.

1. * Corresponding authors.

   E-mail addresses: songyz@sibet.ac.cn (Y. Song), wenfeidong@sibet.ac.cn (W. Dong). [↑](#footnote-ref-1)
2. [↑](#footnote-ref-2)
